# Land Management Legacy Affects Abundance and Function of the *acdS* Gene in Wheat Root Associated Pseudomonads

**DOI:** 10.3389/fmicb.2021.611339

**Published:** 2021-10-27

**Authors:** Heather L. Ruscoe, Rodrigo G. Taketani, Ian M. Clark, George Lund, David Hughes, Ian C. Dodd, Penny R. Hirsch, Tim H. Mauchline

**Affiliations:** ^1^Sustainable Agriculture Sciences, Rothamsted Research, Harpenden, United Kingdom; ^2^The Lancaster Environment Centre, Lancaster University, Lancaster, United Kingdom

**Keywords:** root, *Pseudomonas*, rhizosphere, wheat, land use intensity, ACC deaminase

## Abstract

Land management practices can vastly influence belowground plant traits due to chemical, physical, and biological alteration of soil properties. Beneficial *Pseudomonas* spp. are agriculturally relevant bacteria with a plethora of plant growth promoting (PGP) qualities, including the potential to alter plant physiology by modulating plant produced ethylene *via* the action of the bacterial enzyme 1-aminocyclopropane-1-carboxylate (ACC) deaminase (*acdS*). This study evaluated the impact of land management legacy on the selection and function of wheat root associated culturable pseudomonad isolates. Three distinct previous land uses prior to wheat culture (grassland, arable, and bare fallow) were tested and culturable pseudomonad abundance, phylogeny (*gyrB* and *acdS* genes), function (ACC deaminase activity), and the co-selection of *acdS* with other PGP genes examined. The pseudomonad community could to some extent be discriminated based on previous land use. The isolates from rhizosphere and root compartments of wheat had a higher *acdS* gene frequency than the bulk soil, particularly in plants grown in soil from the bare fallow treatment which is known to have degraded soil properties such as low nutrient availability. Additionally, other genes of interest to agriculture encoding anti-fungal metabolites, siderophores, and genes involved in nitrogen metabolism were highly positively associated with the presence of the *acdS* gene in the long-term arable treatment in the genomes of these isolates. In contrast, genes involved in antibiotic resistance and type VI secretion systems along with nitrogen cycling genes were highly positively correlated with the *acdS* gene in bare fallow isolated pseudomonad. This highlights that the three land managements prior to wheat culture present different selection pressures that can shape culturable pseudomonad community structure and function either directly or indirectly *via* the influence of wheat roots.

## Introduction

Globally, intensive croplands have been expanding since the 1960s, helping to achieve food security by increasing productivity (Agren et al., 2013). However, intensive land use has been linked to soil erosion and compaction, altered nutrient cycling, depleted organic matter levels, salinization, acidification, and pollution (Pretty and Shah, 1997; Doran and Zeiss, 2000; European Commission, 2002; Kibblewhite et al., 2008; Muhammed et al., 2018). These impacts can disrupt soil ecosystem services, thereby threatening the sustainability of the food system for future generations (Edwards, 2002). Conversely, low intensity land-uses such as grasslands, scrublands, and forests are associated with promoting these ecosystem services (Cardinale et al., 2012). A diversity of plant species in such systems offer long-term topsoil cover, along with a reliable source of photosynthetically fixed carbon and organic matter inputs compared to high intensity managements (Piccolo et al., 2008). This can increase soil organic carbon (SOC) levels, aggregate stability, soil pore structure, and biodiversity of micro and macrofauna (Ding et al., 2013; Muhammed et al., 2018).

Different land management practices can alter the soil chemical, physical, and biological environment directly, e.g., *via* fertilizer application and tillage, as well as indirectly by influencing plant root traits (Wedin and Tilman, 1996; Berg and Smalla, 2009; Gregory et al., 2016; Boeddinghaus et al., 2019). These can each have dramatic impacts on the soil microbiome (Edwards, 2002; Foley et al., 2005; Cardinale et al., 2012; Allan et al., 2014, 2015; Kavamura et al., 2019). Increases in microbial biomass have been associated with low intensity managements that receive higher C inputs throughout the year compared to high intensity practices that conventionally utilize monocultures, pesticides, tillage and are not under permanent crop cover (Vries et al., 2013; Estendorfer et al., 2017; Hirsch et al., 2017; Li et al., 2018). Increases in microbial community diversity have also been observed for low-moderate intensity land uses compared to high intensity practices (Tardy et al., 2015; Kavamura et al., 2018). Alterations in microbial community structure provide useful indicators of the impacts of agricultural practices, but how such differences in microbial community structure translate into functional shifts is currently not well known. Studies have centered around nutrient cycling with impacts on carbon, nitrogen, and phosphorus cycling across different land-use intensities (Paula et al., 2014; Hirsch et al., 2017; Neal et al., 2017; Wang et al., 2018; Perez-Brandan et al., 2019). How the soil and plant environment affects the ecology of other microbial traits of interest to agriculture such as microbial phytohormones, has been less studied.

Phytohormones allow plants to alter their growth, development, and physiology in response to environmental factors, including many soil properties such as water and nutrient status (Lymperopoulos et al., 2018). Microbes capable of synthesizing or degrading phytohormones can thereby modify plant physiology and are a desirable target for the microbial inoculant industry (Dodd et al., 2010). 1-Aminocyclopropane-1-carboxylate (ACC) deaminase is an enzyme of particular agricultural interest, as microbes with this trait can modulate *in planta* production of the stress-associated phytohormone ethylene (Li et al., 2000). ACC deaminase cleaves ACC, the immediate biosynthetic precursor of ethylene, to release α-ketobutyrate and ammonia. Root-exuded ACC can be utilized by rhizosphere microbes as a C and N source and consequently decrease root (and shoot) ethylene production, thereby ameliorating growth inhibition associated with high levels of ethylene (Pierik et al., 2006; Nascimento et al., 2014). The ACC deaminase structural gene (*acdS*) is enriched in rhizosphere microbial populations when compared to bulk soil and non-cultivated soils (Marasco et al., 2012; Bouffaud et al., 2018) and has greater rhizosphere abundance when plants are grown in water-limited environments (Siddikee et al., 2010; Timmusk et al., 2011; Stromberger et al., 2017). Thus, ACC deaminase could be a potential marker for plant-microbe interactions under stress-associated soil conditions.

Bacterial isolates from the genus *Pseudomonas* are considered important plant growth promoting rhizobacteria (PGPR) (David et al., 2018). Their ability to alleviate environmental stress, produce PGP substances or act as antagonists to plant pathogens is widely explored (Meliani et al., 2017). However, many aspects of its ecology and how environment changes affect its diversity is still unknown. This study focused on the impact of land management on populations of the soil and rhizosphere dwelling model bacterium *Pseudomonas* spp. in terms of abundance, phylogeny (*gyrB* and *acdS* genes), function (ACC deaminase activity) and the co-selection of *acdS* with other PGP genes. The Highfield experiment at Rothamsted Research, United Kingdom, is a long-term experiment which was initially set up to look at the effects on soil properties when converting permanent grassland into arable (1949) and bare fallow (1959) managements. As these practices induced vast soil chemical, physical, and biological differences (see Supplementary Table 1), it provides an ideal opportunity to study the impacts of different land use intensities, independent of soil type and climate on beneficial soil bacteria. Marked declines in C, N, and P levels, aggregate structure, pH, and microbial and mesofaunal abundance are well documented in the bare fallow and arable plots compared to the grassland plots (Coleman et al., 1997; Watts et al., 2001; Hirsch et al., 2009). Better understanding of the distribution of ACC deaminase producing pseudomonads across different land managements may inform farming practices that encourage beneficial soil-plant-microbe interactions. We hypothesized that land management impacts pseudomonad community structure, with the more degraded soil properties of the bare fallow and arable soils indirectly selecting for a higher *acdS* gene abundance in the rhizosphere and root of wheat.

## Materials and Methods

### Soil Sampling and Crop Genotypes

The A horizon of soil was sampled to a depth of 25 cm (discarding the top 2 cm) for each of the continuous plots of grassland, arable and bare fallow managements at the Highfield experiment (51.80420°N, 0.36140°W) using a hand trowel 3 cm diameter corer. Ten soil samples were taken in a “W” formation across each plot; samples for a given soil treatment were then pooled. Next, the mixed soil for each treatment was sieved (2 mm gauge), homogenized and subsequently used in pot experiments with the hexaploid spring wheat *Triticum aestivum* cv. Cadenza. At the time of sampling, arable plots had been under continuous winter wheat (most recently *T. aestivum* cv. Hereward seed coated with an insecticide/fungicide treatment of Redigo/Deter, Bayer CropScience) production, receiving ammonium nitrate fertilization to provide approximately 220 kg-N ha^–1^ y^–1^, with an additional 250 kg-K ha^–1^ and 65 kg-P ha^–1^ every three years since 1949. The bare fallow plots had been maintained crop- and weed-free by regular tilling and the occasional use of herbicide since 1959. Grassland plots had been maintained as a sward of mixed grasses for over 200 years and mowed twice during summer months.

### Pot Cultivation of Wheat

Seeds were surface sterilized (75% ethanol wash for 30 s followed by 3% sodium hypochlorite for 10 min and rinsed thoroughly with sterile distilled water) and pre-germinated on filter paper soaked in sterile distilled water in Petri dishes. After 36 h, single pre-germinated seeds were sown in 9 cm diameter × 10 cm height pots containing around 200 g of Highfield soil from the three different managements, with five replicates per treatment. Pots were incubated in the glasshouse at 20°C with a 16-h per day light regime, and were watered daily with tap water.

### Sampling of the Rhizosphere and Root (Rhizoplane and Endosphere)

Rhizosphere soil was sampled at the wheat early flowering stage (61 Zadoks) by breaking away the bulk soil to reveal soil closely adhering to the root system, which was removed from the roots by gently dislodging into a sterile tube. To assess pseudomonad communities more closely associated with wheat, the rhizoplane and endosphere were treated as one sample and referred to hereafter as the root compartment. After collecting the rhizosphere soil, unwashed roots were cut with 70% ethanol sterilized scissors and ground using a mortar and pestle at a ratio of 1 g root to 9 mL sterile water.

### Soil Dilutions and Isolation of *Pseudomonas* spp.

Homogenized rhizosphere soil and bulk soil samples (1 g) were diluted separately in 9 mL of sterile distilled water followed by vortex mixing for 10 min to ensure bacterial cells were dislodged from soil particles or the root and in suspension. Serial dilutions were carried out to a dilution factor of 10^–5^ for bulk soil and rhizosphere samples and 10^–7^ for root samples. Next, 100 μL of suspensions were spread onto Petri dishes with *Pseudomonas* Selective agar (PSA, Oxoid^*TM*^) supplemented with Cetrimide, Fucidin, and Cephalosporin (CFC, Oxoid^*TM*^), as per the manufacturer’s instructions, to select against fungi and other bacterial species. The plates were incubated at 28°C for 24--48 h. Random selection using the online random number generator^1^ was used to select isolates from each treatment for subsequent creation of a *Pseudomonas* culture collection. Six isolates from each of five replicates per niche (bulk soil, rhizosphere, and root) were randomly selected for further analysis, totaling 270 isolates.

### Identifying Isolates That Utilize 1-Aminocyclopropane-1-Carboxylate as a Sole N Source

Isolates were screened for their ability to use ACC as a sole nitrogen source following the method described in Penrose and Glick (2003). Bacterial isolates were grown overnight in LB broth at 150 rpm at 28°C. Cultures were adjusted to an Optimal Density (OD) of 1.0 at an absorbance of 600 nm using a spectrophotometer, before transferring 1 mL of culture into 50 mL Dworkin and Foster (DF) minimal medium with ammonium sulfate as a nitrogen (N) source and grown for 24 h, shaking at 150 rpm at 28°C. DF media was prepared as follows (per liter): 4.0 g KH_2_PO_4_, 6.0 g Na_2_HPO_4_, 0.2 g MgSO_4_⋅7H_2_O, 2.0 g glucose, 2.0 g gluconic acid, and 2.0 g citric acid with trace elements: 1 mg FeSO_4_⋅7H_2_O, 10 μg H_3_BO_3_, 11.19 μg MnSO_4_⋅H_2_O, 124.6 μg ZnSO_4_⋅7H_2_O, 78.22 μg CuSO_4_⋅5H_2_O, 10 μg MoO_3_, in addition to 2.0 g (NH_4_)_2_SO_4_ as a sole N source. The medium was adjusted to pH 7.2.

After 24 h, cultures were centrifuged at 5,000 rpm, the supernatant was discarded, and cells washed twice in DF medium without a N source. Cells suspended in N free DF medium (1 mL) were transferred to 25 mL of DF + 3.0 mM ACC (Sigma-Aldrich) as the sole N source. DF + ACC medium was prepared from a 0.5 M stock solution of ACC, which was filter-sterilized through a 0.2 μm membrane, the filtrate was collected and frozen at −20°C for storage. Prior to inoculation, the ACC solution was thawed and a 150 μL aliquot was added to the sterile DF minimal medium in place of (NH_4_)_2_SO_4_. Following inoculation, the culture was grown at 150 rpm for 24 h at 28°C. Isolates that did not grow in the DF + ACC medium were regarded as not having a functional ACC deaminase enzyme.

### Calibration Curve for Quantifying 1-Aminocyclopropane-1-Carboxylate Consumption

For isolates that tested positive for ACC deaminase activity, their ACC consumption was assessed using a colorimetric ninhydrin assay as described by Li et al. (2015). A total of 500 mg of ninhydrin and 15 mg of ascorbic acid were dissolved in 60 mL of ethylene glycol and mixed with 60 mL of 1M citrate buffer (pH 6.0) prior to use. Citrate buffer was prepared with 12.04 g sodium citrate dihydrate and 11.34 g citric acid in 1 L of distilled water and adjusted to pH 6.0.

A standard colorimetric calibration curve was performed with varying ACC concentrations. DF medium was prepared with respective ACC working concentrations of 3 mM, 2.5 mM, 2 mM, 1.5 mM, 1 mM, 0.5 mM, and 0 mM. Each ACC working solution (1 mL) was mixed with 2 mL of ninhydrin reagent in glass test tubes, which were capped, shaken and placed in a boiling water bath (100°C). After 15 min, the tubes were moved into a water bath at room temperature (∼20°C) for 2 min. The samples were then shaken for 30 s and left to stand at room temperature for 10 min to allow purple coloration to develop. The solution was transferred into a cuvette and absorbance measured at 570 nm with a Varioskan^®^ spectrophotometer (Thermo Scientific^*TM*^).

### Screening Isolates for 1-Aminocyclopropane-1-Carboxylate Consumption

To test the bacterial isolates, cultures grown in DF + ACC medium were centrifuged at 5,000 rpm for 5 min and a 1 mL supernatant of bacterial culture was added to 2 mL of ninhydrin reagent in glass test tubes, which were capped, shaken and placed in a boiling water bath (100°C). After 15 min, the tubes were moved into a water bath at room temperature (∼20°C) for 2 min. The samples were shaken for 30 s and left to stand at room temperature for 10 min before transferring to a cuvette and absorbance measured at 570 nm by a spectrophotometer.

### DNA Release/Extraction

MicroLYSIS^®^-PLUS (Microzone Ltd., Haywards Heath, West Sussex, United Kingdom) was used to release DNA from single colonies of each of the ACC positive and 18 ACC negative isolates following the manufacturer’s protocol and utilized as the template DNA in polymerase chain reaction (PCR) reactions for amplifying the and *acdS* gene fragments. These pseudomonad isolates were selected for full genome sequencing, in which high quality genomic DNA was extracted from 10 mL LB cultures of each isolate using the GenElute genomic DNA extraction kit (Sigma-Aldrich), as per the manufacturer’s protocol. DNA quantity was determined using the Qubit Fluorometric Quantification (Life Technologies) using the manufacturer’s instructions. DNA quality was determined using the NanoDrop Microvolume UV spectrophotometer (Thermo Fisher Scientific Inc.) by OD at 260 nm, the 260/280 ratio was used to determine DNA quality in addition to running the samples on a 1.5% (w/v) agarose gel in 1 × Tris-borate-EDTA (TBE) stained with EtBr (0.2 μg mL^–1^) with TBE as the running buffer. Bands of DNA were viewed under UV light to visually detect smears arising from any degraded DNA.

### Polymerase Chain Reaction

Each PCR mixture was 25 μL in total and consisted of 10 × Bioline reaction buffer, Bioline dNTP mix 25 mM each, Bioline MgCl_2_ 50 mM, forward and reverse primers (both at 0.1 μM), BioLine DNA polymerase (0.25 μL), microLYSIS^®^ Plus DNA extract as a template (1 μL) and 18 μL of nuclease-free water. PCR products (5 μL) were examined on a 1.5% (w/v) agarose gel and gene products purified using the MinElute^®^ PCR purification kit (Qiagen) according to the manufacturer’s instructions. Degenerate primers and PCR programs for amplification of *acdS* were as follows – Forward (acdSf3): ATCGGCGGCATCCAGWSNAAYCANAC (26 bp) (Li et al., 2015); Reverse (acdSr3) GTGCATCGA CTTGCCCTCRTANACNGGRT (23 bp) (Li et al., 2015); 94°C for 2 min followed by 35 cycles at 94°C for 45 s, 60°C for 30 s, and 72°C for 1 min and a final elongation of 72°C for 5 min.

### DNA Purification and Quantification of Polymerase Chain Reaction Products

Polymerase chain reaction products were examined on 1.5% (w/v) 1 × TBE gels stained with EtBr (0.2 μg mL^–1^) and with 1 × TBE as the running buffer. Bands of DNA were viewed under UV light to identify fragments of the correct size which were then purified using the MinElute PCR purification kit (Qiagen) according to the manufacturer’s guidelines. Purified DNA was then quantified using the NanoDrop^*TM*^ spectrophotometer ND-1500 (Labtech) following the manufacturer’s protocol.

### Genome Sequencing

Genome sequencing for each of the *acdS* positive isolates and 18 randomly selected *acdS* negative isolates (6 from each soil treatment) was performed by Microbes NG with × 30 sequence coverage.^2^ A total of 30 μL of DNA at a concentration of 50 ng μL^–1^ L was sent to Microbes NG, where sequencing libraries for the samples were prepared. Libraries were sequenced on an Illumina HiSeq using a 250 bp paired end protocol. Reads were adapter trimmed using Trimmomatic 0.30 with a sliding window quality cut-off of Q15. *De novo* assembly was performed on samples using SPAdes version 3.7 (Nurk et al., 2013) and contigs were annotated using Prokka 1.11 (Seemann, 2014). Genome analysis was initially performed by searching for PGP genes of interest within the Prokka annotations received from microbesNG – see Supplementary Table 2 for a summary of genes searched for. The genomes were uploaded to the Rapid Annotation using Subsystem Technology (RAST) pipeline for additional annotations (Aziz et al., 2008). If genes of interest were not found in the annotated genomes, sequences were download from NCBI and searched for using Basic Local Alignment Search Tool (BLAST) within the RAST pipeline (Aziz et al., 2008). The DNA gyrase subunit B gene (*gyrB*) sequences were detected and retrieved from the sequenced genomes using the BLAST approach. The obtained genomes had an average 6,536,076 bp (ranging from 5.93 and 7.05 Mb) and 185 contigs with N50 of 118,823 (see Supplementary Table 3).

### DNA Sequence Analysis of the *gyrB* and *acdS* Genes

The *acdS* and *gyrB* gene sequences were retrieved from the annotated genomes and imported into Geneious. Sequences for both *gyrB* and *acdS* genes were multi-aligned using the software MUSCLE and subsequently trimmed to result in a sequence length of 879 bp for each of the *gyrB* sequences and 1,150 bp for the *acdS* sequences. Before phylogenetic construction, the J model test (2.1.10) was used to determine the best model fit for the alignment. Separate maximum likelihood trees for both the *gyrB* and *acdS* gene were then constructed utilizing a transitional model (TIM 012032) with 1,000 bootstraps using the software PhyML (Guindon et al., 2010). The NCBI BLAST tool^3^ was used to compare sequences from individual isolates to the NCBI database.

### *In silico* Analysis of the *acdS* Gene

Nucleotide sequences for the structural (*acdS)* and regulatory (*acdR*) ACC deaminase gene were extracted from the annotated genomes. The amino acids Lys51, Ser78, Tyr294, Glu295, and Leu322 have all been implicated as essential for the functioning of the *acdS* gene in the reference isolate *Pseudomonas* sp. UW4. Nucleotides were translated into amino acid sequences followed by a multiple alignment with the amino acid sequence from the reference *Pseudomonas* sp. UW4 and the presence of the above amino acids was deemed necessary to score for a putative *acdS* gene. The *Pseudomonas* sp. UW4 ACC deaminase amino acid sequence utilized was obtained from the NCBI database.

### Statistical Analyses

Five replicate pots were used for each of the soil management treatments (*n* = 5). One-way ANOVA compared mean CFU across the different treatments. When data did not follow a normal distribution, the results were LOG10 transformed before statistical analysis. Any significant results were followed by the *post hoc* Tukey analysis, to identify significantly different treatment effects in the software R studio. For gene presence and absence analysis, each strain was given a value of 1 (gene present) or 0 (gene absent). Where no variation was observed in gene presence between all of the isolates, genes were removed prior to conducting the correlation analysis. Correlation analyses were computed using the Spearman’s rank method in Microsoft^®^ Excel^®^.

## Results

### *Pseudomonas* spp. Core Community Structure

**FIGURE 1:**
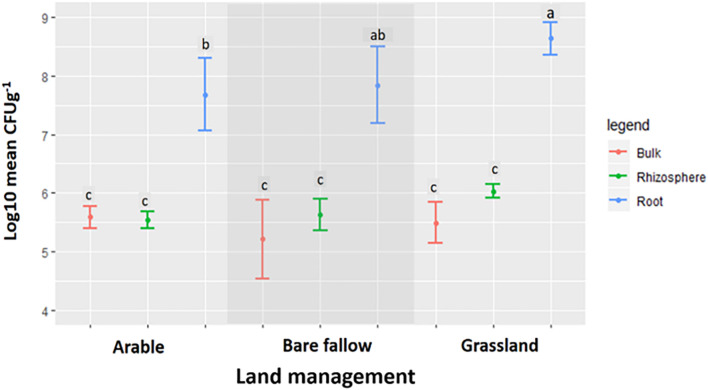
The abundance of *Pseudomonas* spp. isolated from bulk soil, the rhizosphere, and roots of wheat grown in soils from three different land managements. Log transformed data for the mean CFU g^–1^ ± SD of *Pseudomonas* spp. isolated from bulk soil, the rhizosphere, and the roots of wheat grown in soils from arable, grassland, and bare fallow managed soils. Symbols sharing the same letter are not significantly (*P* < 0.05) different according to Tukey’s HSD *post hoc* test.

**FIGURE 2:**
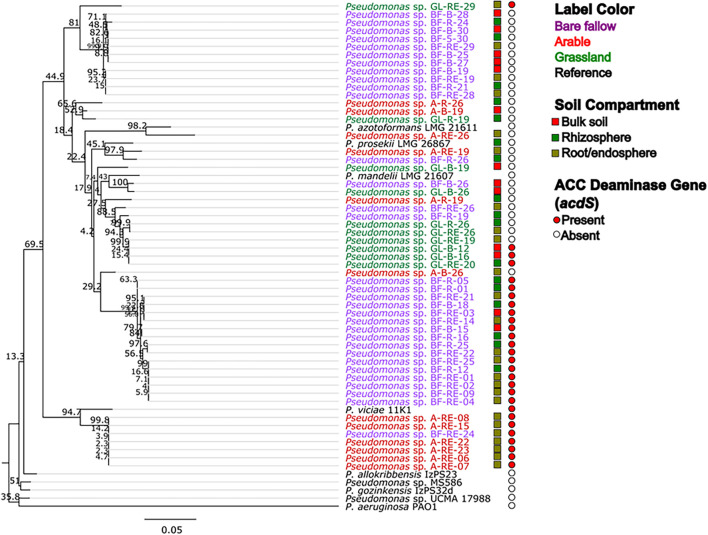
Maximum likelihood (ML) tree based on *gyrB* nucleotide sequences from genome sequenced *Pseudomonas* strains associated with wheat grown in soils from three different land managements. The 54 isolates from bulk soil, rhizosphere soil, and roots of wheat grown in three distinct land management regimes (bare fallow, arable, and grassland) were phylogenetically analyzed utilizing a ML tree with a transitional model. Percentage bootstrap are indicated at branching nodes. Branch labels are colored to indicate the soil management where the bacteria was isolated and boxes beside labels indicate the soil compartment. Circles indicate the presence or absence of *acdS* gene in the genome of the isolate.

Across all three land managements (Figure 1), abundance of *Pseudomonas* spp. was similar in bulk soil and rhizosphere communities (ranging from 10^5^ to 10^6^ mean CFU g^–1^) whilst pseudomonads associated with wheat roots were significantly more abundant (10^7^–10^8^ mean CFU g^–1^ root). All 270 bacterial isolates were identified as belonging to the *Pseudomonas* genus by Sanger sequencing analysis of the *gyrB* gene (data not shown). Phylogenetic analysis of the *gyrB* gene from the genome sequenced isolates (Figure 2) revealed that pseudomonad communities could be partially resolved according to previous land use. Isolates derived from the previously grassland managed soil were primarily represented in one clade most similarly related to *Pseudomonas mandelii* LMG 21607. Those from the arable managed soil being present in a wide range of clades across the tree, with one clade being associated with the *Pseudomonas viciae* reference sequence. Two clades of closely related isolates were identified for pseudomonads cultured from the bare fallow treatment, one being dominated by bulk soil isolates and the other root associated isolates. Neither of these clades were closely related to any of the reference *Pseudomonas* species included in the tree. In addition, a diverse clade was observed which possessed isolates from all previous land uses as well as the reference accessions representative of *Pseudomonas prosekii* and *Pseudomonas azotoformans*. These observations highlight that both land management and niche play a role in structuring pseudomonad communities.

### 1-Aminocyclopropane-1-Carboxylate Deaminase Gene Distribution and Phylogeny

**FIGURE 3:**
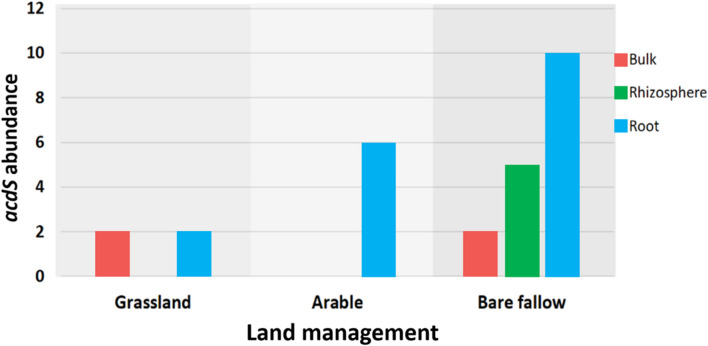
The distribution of the *acdS* gene abundance across three different land managements. A total of 27 isolates possessed the *acdS* gene, the bar graph showing its abundance across the different niche compartments of bulk soil, rhizosphere soil, and wheat roots.

**FIGURE 4:**
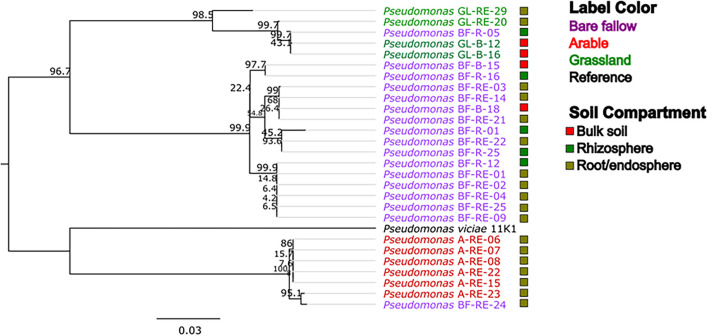
Phylogeny of the *acdS* gene in pseudomonads from three different land managements. *acdS* sequences from bulk soil, rhizosphere, and roots associated with wheat grown in three distinct land management regimes (bare fallow, arable, and grassland) were phylogenetically analyzed utilizing a ML tree with a transitional model and 1,000 bootstraps in the software PhyML. Percentage bootstrap are indicated at branching nodes. Branch labels are colored to indicate the soil management where the bacteria was isolated and boxes beside labels indicate the soil compartment.

To better understand possible functional differences relating to pseudomonad *gyrB* phylogeny associated with the three land managements, isolates were screened for the presence of the ACC deaminase structural gene *acdS*, with an expected size of ∼680 bp. While the ACC deaminase PCR screen identified 36 isolates with the *acdS* gene, subsequent WGS analysis identified only 27 isolates (9.6% of the culture library) possessing the gene (Figure 2, see also Supplementary Table 3 for isolate information). Lack of specificity of PCR primers likely overestimated the presence of the *acdS* gene in isolates. The previously bare fallow soil had the highest (17 isolates) abundance of *acdS* isolates, followed by the continuous arable (six isolates) and the previously grassland soil (four isolates). The isolates which possessed the *acdS* gene came from a range of niche compartments, though most were isolated from wheat roots (Figure 3). Also, the majority of those with close phylogenetic relationship were cultured from different compartments or from plants cultured in different pots (Figure 2 and Supplementary Table 4). As such it is unlikely that the isolates are clonal, but rather the trends observed are due to biotic and abiotic selection factors. A potential “rhizosphere effect” was observed in the previously bare fallow soil, with abundance of the *acdS* gene increasing in number from the bulk soil to the rhizosphere and increasing further again in the root compartment. The regulatory *acdR* gene, which is essential for the functioning of ACC deaminase, was found in the genomes of each of these isolates. The *acdS* phylogeny (Figure 4) reveals distinct clades associated with land management, with the isolates derived from the arable soil management being most closely related to the reference strain *P. viciae* 11K1.

### 1-Aminocyclopropane-1-Carboxylate Deaminase Functionality

**FIGURE 5:**
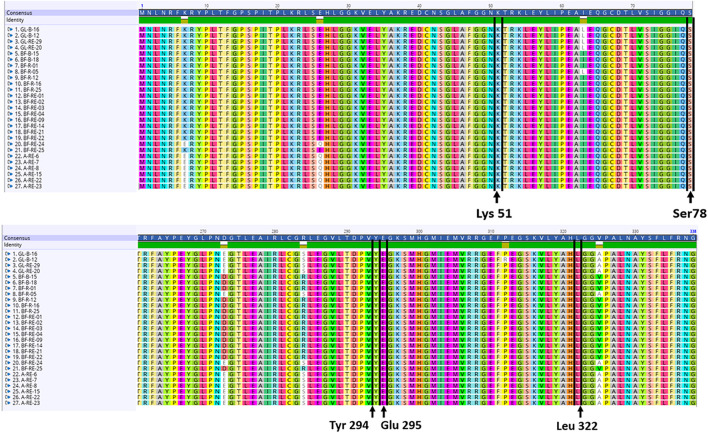
*In silico* analysis of the *acdS* translated amino acid sequences. The amino acids Lys51, Ser78, Tyr294, Glu295, and Leu322 have all been implicated as essential for the functioning of the *acdS* gene in the reference isolate *Pseudomonas* sp. UW4. The alignments shown here with amino acid sequences translated from the *acdS* gene were each found to have all 5 amino acids in the same position as the reference isolate *Pseudomonas* sp. UW4.

**FIGURE 6:**
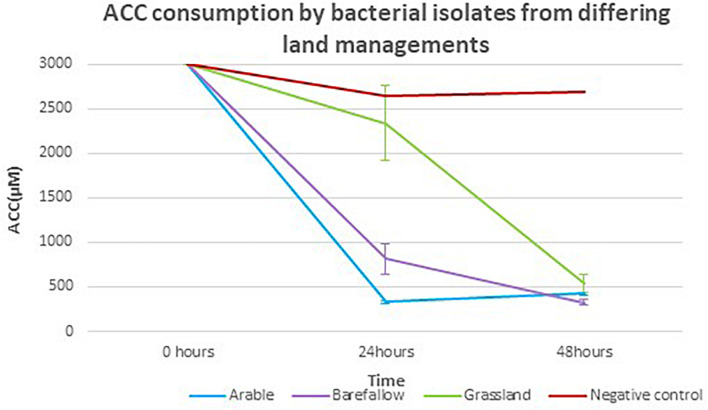
1-Aminocyclopropane-1-carboxylate consumption by isolates with the *acdS* gene grown in media with ACC as the sole nitrogen source. The mean ACC consumption for isolates from arable, bare fallow, and grassland treatments, grown in Dworkin and Foster (DF) + ACC medium with ACC concentration quantified by a ninhydrin colorimeter test at 24 and 48 h post inoculation.

Each of the 27 isolates identified as having the *acdS* gene from WGS had the key amino acid residues Lys51, Ser78, Tyr294, Glu295, and Leu322, all of which have been implicated as essential for functioning of the *acdS* gene in the reference isolate *Pseudomonas* sp. UW4 (Figure 5). *In silico* sequence analysis identified amino acid differences at 13 positions in the *acdS* gene. In addition to *in silico* analysis of the *acdS* gene, each of the *acdS* positive isolates grew in DF medium with ACC as the sole nitrogen source, whilst a negative control isolate without the *acdS* gene did not grow. Thus, all *acdS* positive pseudomonads had the functional potential to lower plant ethylene emission. All *acdS* positive isolates had substantially lower ACC in the growth medium after 48 h, whilst the negative control had levels of ACC close to the starting levels of 3 mM (Figure 6).

**FIGURE 7:**
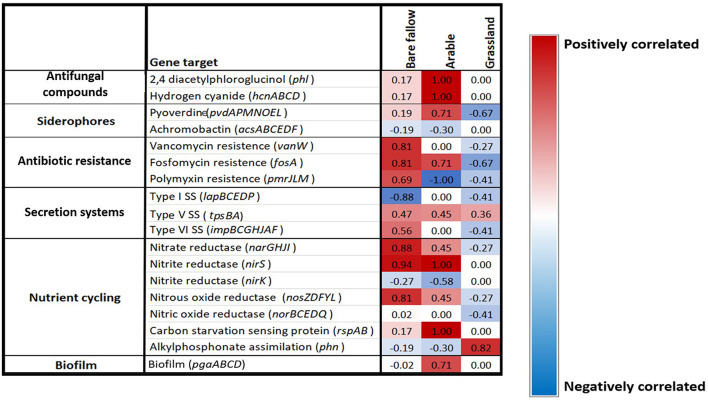
Gene presence/absence correlation coefficient analysis. Spearman’s rank correlation coefficients between the presence or absence of a selection of PGPR traits correlated against the presence of the *acdS* gene in bacterial isolates from three distinct land managements. The scale range is from dark blue (highly negatively correlated) to bright red (highly positively correlated), light colors represent low correlations.

To determine if the ACC deaminase gene was co-selected with other traits of interest to agriculture across the three previous land managements, a Spearman’s rank coefficient correlation analysis was performed against the *acdS* negative and *acdS* positive isolates to determine any correlations. Interestingly, the ACC deaminase gene appeared to be co-selected with different genes according to land management. Genes involved in anti-fungal metabolite, siderophore, and biofilm production as well as N cycling genes were highly positively correlated with the *acdS* gene in the continuous arable treatment. Whilst genes involved in antibiotic resistance and type VI secretion systems, along with N cycling genes were highly positively correlated with the *acdS* gene in the previously bare fallow treatment. However, these genes were negatively correlated with the *acdS* gene in the grassland soil whilst alkylphosphonate assimilation was the only gene studied that appeared to be highly positively correlated with the *acdS* gene in this soil management type (Figure 7). Type V secretion system genes were positively correlated with the *acdS* gene across all three land managements.

## Discussion

### Land Management and Pseudomonad Community Structure

The isolation of bacterial cultures is known for its selection biases which prompted the growth of culture-independent approaches in the past three decades. However, culturing has allowed this study an in-depth look into the genetic potential and diversity of *Pseudomonas* spp. from different soil niches. Land management is known to affect soil biodiversity and biomass, but how it impacts community dynamics of plant associated members such as *Pseudomonas* spp. is not well understood. *Pseudomonas fluorescens* isolates are commonly reported as plant associated bacteria found in the rhizosphere and roots, due to their ability to metabolize various carbon sources released in root exudates (Hol et al., 2013). As expected, our data showed that the root compartment of wheat grown in soil regardless of land use history supported 100–1,000-fold higher abundance of pseudomonads than bulk and rhizosphere soil. Interestingly, pseudomonad abundance levels on plate counts were similar across rhizosphere and bulk soil communities, which highlights that wheat roots played a strong role in driving pseudomonad selection in this study. Despite the different bias of the methods, comparable findings using culture independent techniques based on the 16S rRNA gene, have shown that the *Pseudomonadaceae* are enriched in the root endosphere (Estendorfer et al., 2017; Kavamura et al., 2019). The low intensity grassland soil, which we used to cultivate wheat in for the first time in at least 200 years, was associated with a greater abundance of pseudomonads in the wheat root compartment compared to previously bare fallow and continuous arable plots, despite a similar abundance being found in the bulk and rhizosphere compartments. Higher microbial biomass has been associated with low intensity land uses such as forests and pastures when compared to high intensity agricultural practices, although these previous studies did not focus on pseudomonad communities (Zhang et al., 2016; Van Leeuwen et al., 2017).

Analysis of the *gyrB* gene revealed differences in pseudomonad isolates phylogeny associated with wheat grown in soils from the different land use histories. However, no clear difference in pseudomonad diversity was observed according to previous land use. The occurrence of a mixed clade of isolates derived from all previous land uses suggests that phylogenetic diversity is retained in contrasting soil management types and agrees with previous work performed on samples from this site (Hirsch et al., 2009). The observation that isolates derived from bare fallow managed soil formed a bulk soil as well as a root associated clade will be useful for future *in silico* and *in planta* studies with these isolates which could reveal mechanistic insights to niche establishment of pseudomonads in wheat cropping.

### Distribution, Phylogeny, and Function Ability of Isolates With the 1-Aminocyclopropane-1-Carboxylate Deaminase Structural Gene (*acdS*)

The *acdS* gene encoding for ACC deaminase was not a common trait in the pseudomonad communities studied, although it was more abundant in isolates from the bare fallow treatment which accounted for 63% of the total *acdS* isolates identified. Our data indicates that wheat plants appeared to select for the presence of isolates containing the *acdS* gene, with *acdS* positive isolates increasing from bulk to rhizosphere soil with the highest abundance found in the root compartment of plants grown in the previously bare fallow managed soil. A similar enrichment of *acdS* microbes in the rhizosphere compared to bulk soil in other studies further indicates that root exudation of ACC into the rhizosphere potentially selects for microbes that are able to utilize this compound (Bouffaud et al., 2018). Since the bare fallow soil has lower C, N, pH and higher bulk density than the continuous arable and grassland treatments, it presents a more stressful environment for plant growth (Islam et al., 1980; Khan et al., 2015; Neal et al., 2017; Brito et al., 2018). Indeed, previous studies at the Highfield experiment have shown that wheat grown in the previously bare fallow plots had significantly lower grain yields compared to wheat grown in the previously grassland and arable treatments (Hirsch et al., 2017), highlighting that the bare fallow soil presents a more stressful environment for wheat cultivation. Higher selection for *acdS* positive pseudomonad isolates observed here may therefore have occurred in the bare fallow treatment due to greater ACC exudation by wheat roots, providing an additional N and C source in the competitive rhizosphere and root compartment. Albeit, further study is needed to identify specific stressors associated with the soil properties in the bare fallow treatment that may enhance root ACC exudation by wheat.

Interestingly, phylogenetic differences in the *acdS* gene from pseudomonad isolates were observed and sequences clustered according to land management, suggesting that the ACC deaminase gene was influenced *via* different selection pressures related to the three distinct land use histories when cultivating wheat. Amino acid differences were found at 13 different positions in the *acdS* gene across the 27 isolates. In addition, it was found that the isolates which were obtained from wheat plants cultured in the previously grassland soil grew more slowly on glucose and ACC compared to the other isolates. It is not clear if these differences are related to altered efficiency of ACC deaminase activity or due to bare fallow and arable isolates being better adapted for carbon utilization. Since soil type was the same across the different land managements, we suggest that abiotic factors associated with land use intensity, indirectly influenced the selection of *acdS* bacteria to the rhizosphere and root compartment of wheat. More studies are needed to confirm whether root-exuded ACC acts as a potential rhizosphere attractant for *acdS* positive microbes present in the soil environment. Relevantly, soil inoculation with *acdS* positive microbes can decrease plant ACC concentrations in the rhizosphere (Belimov et al., 2015), highlighting the importance of studying *acdS* positive microbes to help elucidate soil-plant-microbe interactions. As all pseudomonads with the *acdS* gene were shown to utilize ACC as a sole nitrogen source, this evidences that the isolates had the functional potential to alter plant ethylene levels. Despite this we did not establish whether the *acdS* gene was being expressed *in situ* and how this may change over the growing period, as sampling only occurred during the wheat flowering stage. As such, more studies into the impacts of land management on soil-plant-microbe interactions at different time points are needed to better understand the temporal dynamics of *acdS* gene abundance and expression.

### Co-selection of the *acdS* Gene With Other Traits of Interest to Agriculture

Since *acdS* bacteria were found in a higher abundance in the rhizosphere and roots of plants grown under stress-associated conditions, the co-selection of other common traits of interest for agriculture was investigated. Interestingly, the presence of the *acdS* gene in pseudomonads isolated from wheat grown in soil from the continuous arable and bare fallow treatments were highly positively correlated with genes associated with biotic stress. Here, genes for anti-fungal metabolite production were found in all *acdS* positive pseudomonads but not in *acdS* negative pseudomonads isolated from the continuous arable treatment. Plant susceptibility to pathogens in intensive monoculture practices is well documented (Piper, 1996; Bellaire et al., 2010; Otani et al., 2019). It is therefore plausible that a higher number of fungal pathogens in the continuous arable plots may have been present compared to the bare fallow and grassland treatments. As these plants did not show any obvious signs of disease, the selection of pseudomonads possessing antifungal metabolite genes may have contributed to prevent the build-up of plant fungal pathogens, akin to a suppressive soil. Although the role of ethylene has been studied in response to biotic stress (Díaz et al., 2002), there are no studies to date that have measured root ACC of plants under such conditions. Further analysis to investigate the presence of well-known pathogens in wheat rhizosphere samples in addition to root phytohormone analysis will help to further unravel these findings.

The *acdS* containing isolates from previously bare fallow soil were highly positively correlated with antibiotic resistance genes, which could suggest that wheat grown in this soil may have been experiencing stress from antagonistic bacteria or fungi. Additionally, type VI secretion system genes were highly correlated with the *acdS* gene in these isolates. Type VI secretion systems have been shown to mediate interactions with a range of Gram-negative bacteria, by translocating effector proteins that can disrupt multiple essential and conserved features in targeted bacteria in a contact-dependent manner (Russell et al., 2014). Collectively, this could highlight the competitive nature of pseudomonads establishing and persisting in the highly dynamic rhizosphere and root compartment, where the ability to consume ACC as a C and N source also provides a colonization advantage.

We showed that isolates from wheat grown in continuous arable and previously bare fallow plots were highly positively correlated with genes involved in N cycling and C starvation genes. Since the arable plots received regular NPK additions prior to the soil being utilized in this study for pot experiments, increases in denitrification genes can be expected, as seen in other studies (Ouyang et al., 2018; Wang et al., 2018). Whilst soil from the bare fallow plots did not receive fertilizer application, they have been shown to have a high bulk density which can facilitate the development of anoxic conditions through decreased pore size (Watts et al., 2001; Gregory et al., 2016). Denitrification is enhanced under such conditions, allowing bacteria to survive by utilizing N as a substitute terminal electron acceptor in place of oxygen in order to respire (Clark et al., 2012). Denitrification can lead to substantial N losses from soil to the atmosphere, potentially reducing the N available for plant uptake. Conversely these traits showed no, or moderately negative correlations with the presence of the *acdS* gene in pseudomonads isolated from wheat grown in the grassland treatment soil. Furthermore, we identified P metabolizing genes belonging to the *pho* gene operon in these isolates. In addition, genes involved in alkylphosphonate assimilation were highly positively correlated with the *acdS* gene in pseudomonads isolated from wheat cultivated in the previously grassland soil, whilst negatively correlated with the isolates from wheat cultivated in continuous arable and bare fallow soil. The arable and bare fallow treatments have previously been shown to have lower organic C and N compared to the grassland treatment (Supplementary Table 1). It is likely that these abiotic factors directly shape pseudomonad communities in soil, in addition to indirectly *via* their influence of plant phenotype. Whilst plant ethylene has been shown as an important regulator of responses to nutrient stress (Tian et al., 2009; Wang et al., 2014), root ACC measurements under nutrient limitations are needed to better understand any possible selection of *acdS* bacteria. Although these results provide an interesting insight into soil–plant–microbe interactions, more studies investigating the link between wheat phytohormone levels, soil properties and microbial communities at different stages of crop development are required to better understand these dynamics.

## Conclusion

To summarize, land management affected culturable pseudomonad community structure in addition to ACC deaminase phylogeny and function. Around 10% of the pseudomonad isolates had the *acdS* gene, however its abundance increased in the plant associated niche compartments of wheat grown in the more degraded bare fallow treatment. Each of the isolated pseudomonads with the *acdS* gene were able to utilize ACC as a sole N source, highlighting the functional potential for these isolates to lower plant ethylene levels. Interestingly, other stress related PGP traits were highly correlated with the presence of *acdS* in isolates from the bare fallow and continuous arable treatment soils, whilst negatively correlated with the *acdS* gene presence in isolates from the grassland soil. In agreement with other studies, this highlights that anthropogenic changes in land use structurally alter soil microbial communities and influence the abundance of functional genes involved in plant-microbe interactions.

## Data Availability Statement

The data presented in the study are deposited in the National Center for Biotechnology Information (NCBI) repository, accession number PRJNA725667 (https://www.ncbi.nlm.nih.gov/bioproject/PRJNA725667).

## Author Contributions

HR, TM, and PH designed the experiments. HR performed the experiments and collected the data. HR, DH, GL, and RT analyzed the data. HR and TM wrote the manuscript. HR, PH, TM, RT, IC, and ID edited and commented on the manuscript.

## Conflict of Interest

The authors declare that the research was conducted in the absence of any commercial or financial relationships that could be construed as a potential conflict of interest.

## Publisher’s Note

All claims expressed in this article are solely those of the authors and do not necessarily represent those of their affiliated organizations, or those of the publisher, the editors and the reviewers. Any product that may be evaluated in this article, or claim that may be made by its manufacturer, is not guaranteed or endorsed by the publisher.

## Footnotes

1
https://www.random.org

2
https://microbesng.uk/

3
https://blast.ncbi.nlm.nih.gov/Blast.cgi
