# Supplementary material for: Land Management Legacy Affects Abundance and Function of the acdS Gene in Wheat Root Associated Pseudomonads
Source: Front Microbiol. 2021 Oct 27;12:611339. doi: 10.3389/fmicb.2021.611339 (PMC8578595; doi:10.3389/fmicb.2021.611339)
Supplement: Supplementary file 1 [file Data_Sheet_1.docx]

**
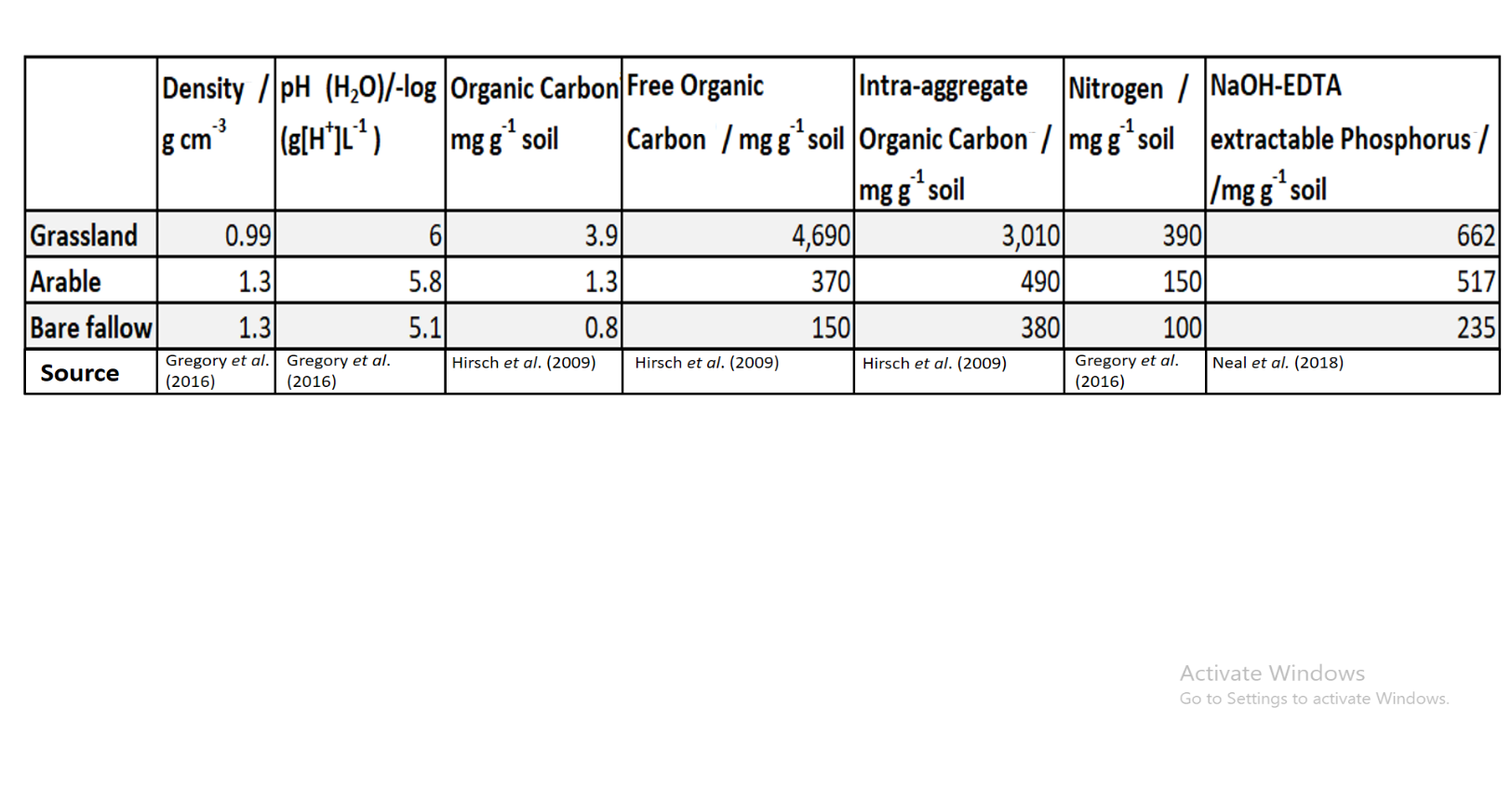
Supplementary data**

**Table S1****. Summary of the physical and chemical properties of soil from the Highfield Ley-Arable experiment**: The table shows chemical and physical measurements of soils taken from the permanent grassland, arable and bare fallow plots of the Highfield experiment. The data shown has been adapted from Neal *et al* 2018 and displays results from Gregory *et al*. (2016), Hirsch *et al*. (2009) and Neal *et al* (2018).

| **Function** | **Target trait** | **Gene name** |
| --- | --- | --- |
| **Bioregulation** | 1-aminocyclopropane-1-carboxylate deaminase | *acdS, acdR* |
|  | Indole-3-acetic acid (auxin) | *iaaMH, nit, nthAB, ipdC* |
|  | tRNA isopentenylpyrophosphate transferase (cytokinin) | *miaA* |
| **Anti-fungal** | Hydrogen cyanide | *hcnAB* |
|  | 2,4-diaceylphloroglucinol | *phlEDBAFGH* |
|  | Phenazine | *phzIRABCDEFGO* |
|  | Pyrrolnitrin | *prnABCD* |
|  | Pyoluteorin | *pltABCDEFGIJKNO* |
| **Siderophore** | Achromobactin | *acsABCEDF* |
|  | Pyochelin | *pchR* |
|  | Pyoverdine | *pvdAPMNOEL* |
|  | Ferrous iron transporter | *efeUOB* |
| **Phosphorous metabolism** | Alkaline phosphatase | *phoA* |
|  | Phosphate regulation | *phoBR* |
|  | Phosphate transport | *pstS* |
|  | Phosphate DING protein | *dinG* |
|  | Glycerophosphodiester phosphodiesterase | *glpQ* |
|  | Phytase | *phyC* |
|  | Glucose dehydrogenase | *gcd* |
| **Nitrogen cycling** | Nitrate reductase | *narGHJI* |
|  | Nitrite reductase | *nirSK* |
|  | Nitrous oxide reductase | *nosZDFYL* |
|  | Nitric oxide reductase | *norBCEDQ* |
| **Abiotic stress tolerance** | Periplasmic glucans | *mdoHGD* |
|  | Glycine betaine synthesis | *betABTCI* |
|  | Carbon starvation protein | *cstA* |
|  | Carbon starvation sensing protein | *rspAB* |
| **Biotic stress tolerance** | Vancomycin resistance | *vanW* |
|  | Fosfomycin resistance | *fosA* |
|  | Polymyxin resistance | *pmrJLM* |
| **Colonisation** | Biofilm | *pgaABCD* |
|  | Flagella structure | *flhAB fliRQ flgDHBC* |
|  | Flagella motor | *motAB filMN* |
|  | Chemotaxis proteins | *cheAVYWV* |
|  | N-acyl-homoserine lactone | *rhlIR lasRI* |
|  | Autoinducer-2 | *isrKRABCDFGPQ* |
|  | Type I SS | *lapBCEDP* |
|  | Type II SS | *tadABCZ* |
|  | Type III SS | *yscU yopB icrD* |
|  | Type IV SS (pilus) | *pilABCTQMNOPE* |
|  | Type IV SS (Fimbria) | *pilY1XVWR fimU* |
|  | Type V SS | *tpsBA* |
|  | Type VI SS | *impBCGHJAF* |

**Table S2. Target genes for screening the genome of *Pseudomonas* spp:** Various genes of interest were screened for across 54 pseudomonad genomes including PGPR traits involved in bioregulation, anti-fungal activity, siderophore production, Nutrient cycling, abiotic stress tolerance, biotic stress tolerance and genes related to rhizosphere colonisation.

| Isolate Code | Length(bp) | #contigs | N50 |
| --- | --- | --- | --- |
| A-B-19 | 6515109 | 120 | 185696 |
| A-B-26 | 6477144 | 130 | 232524 |
| A-R-19 | 7022807 | 201 | 94878 |
| A-R-26 | 6302608 | 162 | 133521 |
| A-RE-15 | 6778838 | 173 | 105546 |
| A-RE-19 | 6310608 | 274 | 180099 |
| A-RE-22 | 6787619 | 167 | 123667 |
| A-RE-23 | 6905894 | 152 | 153582 |
| A-RE-26 | 5937628 | 113 | 155040 |
| A-RE-6 | 6770954 | 206 | 69263 |
| A-RE-7 | 6775839 | 169 | 108563 |
| A-RE-8 | 6777707 | 199 | 74882 |
| BF-B-15 | 6445447 | 244 | 50001 |
| BF-B-18 | 6690550 | 265 | 46369 |
| BF-B-19 | 6598486 | 170 | 162111 |
| BF-B-25 | 6511970 | 141 | 167833 |
| BF-B-26 | 6741353 | 231 | 86552 |
| BF-B-27 | 6253214 | 160 | 100418 |
| BF-B-28 | 6247948 | 178 | 102629 |
| BF-B-30 | 6299446 | 101 | 131640 |
| BF-R-01 | 6800257 | 255 | 57559 |
| BF-R-05 | 6302781 | 229 | 62304 |
| BF-R-12 | 6374324 | 385 | 32524 |
| BF-R-16 | 6485839 | 342 | 34699 |
| BF-R-19 | 6858872 | 199 | 116327 |
| BF-R-21 | 6263437 | 160 | 119778 |
| BF-R-24 | 6222099 | 163 | 132737 |
| BF-R-25 | 6503295 | 132 | 126221 |
| BF-R-26 | 6572573 | 82 | 374255 |
| BF-R-30 | 6356205 | 102 | 188798 |
| BF-RE-01 | 6363420 | 271 | 47593 |
| BF-RE-02 | 6357918 | 338 | 40544 |
| BF-RE-03 | 6665380 | 417 | 30283 |
| BF-RE-04 | 6370867 | 204 | 68636 |
| BF-RE-09 | 6368974 | 196 | 68574 |
| BF-RE-14 | 6702923 | 225 | 59000 |
| BF-RE-19 | 6225560 | 134 | 142149 |
| BF-RE-21 | 6706620 | 148 | 99360 |
| BF-RE-22 | 6520010 | 155 | 138114 |
| BF-RE-24 | 6623522 | 189 | 139552 |
| BF-RE-25 | 6371407 | 128 | 105261 |
| BF-RE-26 | 6415110 | 140 | 148357 |
| BF-RE-28 | 6589166 | 127 | 183859 |
| BF-RE-29 | 6445740 | 155 | 131917 |
| GL-B-12 | 6739307 | 117 | 125208 |
| GL-B-16 | 7054452 | 165 | 100981 |
| GL-B-19 | 6524961 | 224 | 137527 |
| GL-B-26 | 6541118 | 176 | 104903 |
| GL-R-19 | 6502109 | 134 | 134432 |
| GL-R-26 | 6430994 | 139 | 197045 |
| GL-RE-19 | 6861571 | 168 | 99120 |
| GL-RE-20 | 6869057 | 157 | 80422 |
| GL-RE-26 | 6432708 | 141 | 179099 |
| GL-RE-29 | 6374364 | 118 | 144472 |
| Minimum | 5937628 | 82 | 30283 |
| Maximum | 7054452 | 417 | 374255 |
| Average | 6536076 | 185 | 118823 |

**Table S3: *Pseudomonas* spp. genome assembly data.**

| Isolate code | Pot number* | Soil | Niche compartment | *gyr*B NEAREST TYPE STRAIN (ACCESSION NUMBER)** |
| --- | --- | --- | --- | --- |
| A-B-26 | 5 | Arable | Bulk_soil | *Pseudomonas mandelii* LMG 21607 (LT629796.1) |
| A-B-19 | 4 | Arable | Bulk_soil | *Pseudomonas mandelii* LMG 21607 (LT629796.1) |
| A-R-26 | 5 | Arable | Rhizosphere | *Pseudomonas mandelii* LMG 21607 (LT629796.1) |
| A-R-19 | 4 | Arable | Rhizosphere | *Pseudomonas mandelii* LMG 21607 (LT629796.1) |
| A-RE-26 | 5 | Arable | Root | *Pseudomonas viciae* 11K1 (CP035088.1) |
| A-RE-19 | 4 | Arable | Root | *Pseudomonas prosekii* LMG 26867 (LT629762.1) |
| BF-B-26 | 5 | Bare_Fallow | Bulk_soil | *Pseudomonas viciae* 11K1 (CP035088.1) |
| BF-B-19 | 4 | Bare_Fallow | Bulk_soil | *Pseudomonas viciae* 11K1 (CP035088.1) |
| BF-R-26 | 5 | Bare_Fallow | Rhizosphere | *Pseudomonas azotoformans* LMG 21611 (LT629702.1) |
| BF-R-19 | 4 | Bare_Fallow | Rhizosphere | *Pseudomonas viciae* 11K1 (CP035088.1) |
| BF-RE-26 | 5 | Bare_Fallow | Root | *Pseudomonas viciae* 11K1 (CP035088.1) |
| BF-RE-19 | 4 | Bare_Fallow | Root | *Pseudomonas viciae* 11K1 (CP035088.1) |
| GL-B-26 | 5 | Grassland | Bulk_soil | *Pseudomonas mandelii* LMG 21607 (LT629796.1) |
| GL-B-19 | 4 | Grassland | Bulk_soil | *Pseudomonas mandelii* LMG 21607 (LT629796.1) |
| GL-R-26 | 5 | Grassland | Rhizosphere | *Pseudomonas* sp. MS586 (CP014205.2) |
| GL-R-19 | 4 | Grassland | Rhizosphere | *Pseudomonas* sp. UCMA 17988 (MT080625.1) |
| GL-RE-26 | 5 | Grassland | Root | *Pseudomonas mandelii* LMG 21607 (LT629796.1) |
| GL-RE-19 | 4 | Grassland | Root | *Pseudomonas* sp. UCMA 17988 (MT080625.1) |
| BF-B-30 | 5 | Bare_Fallow | Bulk_soil | *Pseudomonas* sp. UCMA 17988 (MT080625.1) |
| BF-R-21 | 4 | Bare_Fallow | Rhizosphere | *Pseudomonas* sp. UCMA 17988 (MT080625.1) |
| BF-R-24 | 4 | Bare_Fallow | Rhizosphere | *Pseudomonas mandelii* LMG 21607 (LT629796.1) |
| BF-R-25 | 5 | Bare_Fallow | Rhizosphere | *Pseudomonas mandelii* LMG 21607 (LT629796.1) |
| BF-R-30 | 5 | Bare_Fallow | Rhizosphere | *Pseudomonas gozinkensis* IzPS32d (CP062253.1) |
| BF-RE-21 | 4 | Bare_Fallow | Root | *Pseudomonas mandelii* LMG 21607 (LT629796.1) |
| BF-RE-22 | 4 | Bare_Fallow | Root | *Pseudomonas mandelii* LMG 21607 (LT629796.1) |
| BF-RE-24 | 4 | Bare_Fallow | Root | *Pseudomonas* sp. MS586 (CP014205.2) |
| BF-RE-25 | 5 | Bare_Fallow | Root | *Pseudomonas* sp. UCMA 17988 (MT080625.1) |
| BF-RE-28 | 5 | Bare_Fallow | Root | *Pseudomonas mandelii* LMG 21607 (LT629796.1) |
| BF-RE-29 | 5 | Bare_Fallow | Root | *Pseudomonas prosekii* LMG 26867 (LT629762.1) |
| A-RE-22 | 4 | Arable | Root | *Pseudomonas* sp. UCMA 17988 (MT080625.1) |
| A-RE-23 | 4 | Arable | Root | *Pseudomonas gozinkensis* IzPS32d (CP062253.) |
| GL-RE-29 | 5 | Grassland | Root | *Pseudomonas gozinkensis* IzPS32d (CP062253.) |
| GL-B-12 | 2 | Grassland | Bulk_soil | *Pseudomonas mandelii* LMG 21607 (LT629796.1) |
| GL-B-16 | 3 | Grassland | Bulk_soil | *Pseudomonas gozinkensis* IzPS32d (CP062253.) |
| GL-RE-20 | 4 | Grassland | Root | *Pseudomonas gozinkensis* IzPS32d (CP062253.) |
| A-RE-6 | 1 | Arable | Root | *Pseudomonas mandelii* LMG 21607 (LT629796.1) |
| A-RE-7 | 2 | Arable | Root | *Pseudomonas* sp. MS586 (CP014205.2) |
| A-RE-8 | 2 | Arable | Root | *Pseudomonas mandelii* LMG 21607 (LT629796.1) |
| A-RE-15 | 3 | Arable | Root | *Pseudomonas mandelii* LMG 21607 (LT629796.1) |
| BF-B-15 | 3 | Bare_Fallow | Bulk_soil | *Pseudomonas viciae* 11K1 (CP035088.1) |
| BF-B-18 | 3 | Bare_Fallow | Bulk_soil | *Pseudomonas gozinkensis* IzPS32d (CP062253.) |
| BF-R-01 | 1 | Bare_Fallow | Rhizosphere | *Pseudomonas mandelii* LMG 21607 (LT629796.1) |
| BF-R-05 | 1 | Bare_Fallow | Rhizosphere | *Pseudomonas* sp. MS586 (CP014205.2) |
| BF-R-12 | 2 | Bare_Fallow | Rhizosphere | *Pseudomonas* sp. UCMA 17988 (MT080625.1) |
| BF-R-16 | 3 | Bare_Fallow | Rhizosphere | *Pseudomonas mandelii* LMG 21607 (LT629796.1) |
| BF-RE-01 | 1 | Bare_Fallow | Root | *Pseudomonas mandelii* LMG 21607 (LT629796.1) |
| BF-RE-02 | 1 | Bare_Fallow | Root | *Pseudomonas mandelii* LMG 21607 (LT629796.1) |
| BF-RE-03 | 1 | Bare_Fallow | Root | *Pseudomonas mandelii* LMG 21607 (LT629796.1) |
| BF-RE-04 | 1 | Bare_Fallow | Root | *Pseudomonas mandelii* LMG 21607 (LT629796.1) |
| BF-RE-09 | 2 | Bare_Fallow | Root | *Pseudomonas mandelii* LMG 21607 (LT629796.1) |
| BF-RE-14 | 3 | Bare_Fallow | Root | *Pseudomonas mandelii* LMG 21607 (LT629796.1) |
| BF-B-25 | 5 | Bare_Fallow | Bulk_soil | *Pseudomonas mandelii* LMG 21607 (LT629796.1) |
| BF-B-27 | 5 | Bare_Fallow | Bulk_soil | *Pseudomonas mandelii* LMG 21607 (LT629796.1) |
| BF-B-28 | 5 | Bare_Fallow | Bulk_soil | *Pseudomonas allokribbensis* IzPS23 (CP062252.1) |

**Table S4: Isolated *Pseudomonas* spp. source information**. * - Pot number refers to which of the 5 different pot replicates the isolate originated. ** - Nearest BlastN match found in the NCBIs’ type strain database.
